# Macroscopic brain gray matter staining: historical protocol overview and neuroanatomy learning applications in second-year medical students

**DOI:** 10.3389/fnana.2023.1227933

**Published:** 2023-08-17

**Authors:** Gustavo Adolfo Villegas-Gomez, Luisa F. Figueredo, A. D. Ramirez, Pedro Jose Quiroga-Padilla, Roberto Rueda-Esteban

**Affiliations:** ^1^Anatomy Section, Universidad de Los Andes School of Medicine, Bogotá, Colombia; ^2^Healthy Brain Aging and Sleep Center, New York University (NYU) Langone Health, New York, NY, United States

**Keywords:** macroscopic staining, brain, Mulligan, Roberts, Prussian Blue, Alston, education

## Abstract

Macroscopic staining in anatomical samples of the central nervous system is a technique that has been used for decades to achieve better differentiation of multiple gray matter structures, such as the cortex, basal ganglia, and cerebellar nuclei. Staining methods are based on using the different components of the brain, mainly the lipids present in the white matter. These techniques have been progressively forgotten while computer renderings are increasing; however, as a primary exposure to surgical anatomy, stained brain specimens are considered a helpful tool. We aim to summarize different staining techniques, their principles, and their current applications for neuroanatomy learning purposes. In total, four gray matter staining protocol descriptions (Mulligan's, Roberts's, Alston's, and Prussian Blue) were performed, as well as Likert scale surveys of second-year medical students about their perceptions of the stained sections. The results showed that the different macroscopic stains for brain tissue are based on lipid and reactant interactions, intending to increase the white matter (WM) and gray matter (GM) contrast. The search also showed that most staining protocols would take 2 days to develop. Efficient preservation options include submerging the sections in formaldehyde solutions, formaldehyde-free solutions, ethanol, or applying plastination techniques. Based on the student's perspective, the stained slices seem to be a valuable alternative to facilitate the study and identification of the basal ganglia and their relationships with the white matter (from 51.2 to 72% based on the Likert scale) compared with the non-stained sections. In conclusion, macroscopic staining of brain tissue continues to be a valuable tool for comprehensively studying the brain. Further research is needed to determine the efficacy of stained specimens as teaching tools.

## 1. Introduction

Historically, differentiation between white matter and gray matter has been a continual development that has sought to improve results in neuroscience learning, teaching, and research (Quester and Schröder, 1997; Savaskan et al., 2009; dos Santos et al., 2019). The discovery of the neuron as a functional unit of the nervous system and the macroscopic study of its division into soma and axons (Delgado-García, 2015) using visible stains gave a twist to the way we understand the brain, its anatomical and physiological division, and the pathologies that affect it (dos Santos et al., 2019). Including color theory in learning also enhances knowledge and attention in a complex task (Mehta and Zhu, 2009). Additionally, specimens began to last longer once different plastination methods were combined with macroscopic staining methods. This aspect is highly relevant today, considering the difficulties in obtaining biological materials and bodies (Savaskan et al., 2009). The use of visible stains in neurosciences is a medical knowledge legacy, which provides high-quality, easily accessible training (Savaskan et al., 2009).

To the best of our knowledge, no previous publications summarize and compare current macroscopic brain gray matter stain technique models while evaluating students' perceptions of their utility in learning neuroanatomy. We aimed to gather and create a historical review of the literature on the most commonly used techniques and their variations through the years. Four staining methods (Mulligan's stain, Prussian Blue stain, Roberts's stain, and Alston's stain) were performed. Learning ease perceptions and perceived utility of stained specimens during a neuroanatomy class were evaluated using a Likert scale survey in a group of second-year medical students. With the results of this article, we highlight the importance of using stained brain sections while teaching neuroanatomy, which could be used as a tool that other training methods can enhance to have a global understanding of the brain's morphology.

## 2. Materials and methods

### 2.1. Literature review

**Figure 1:**
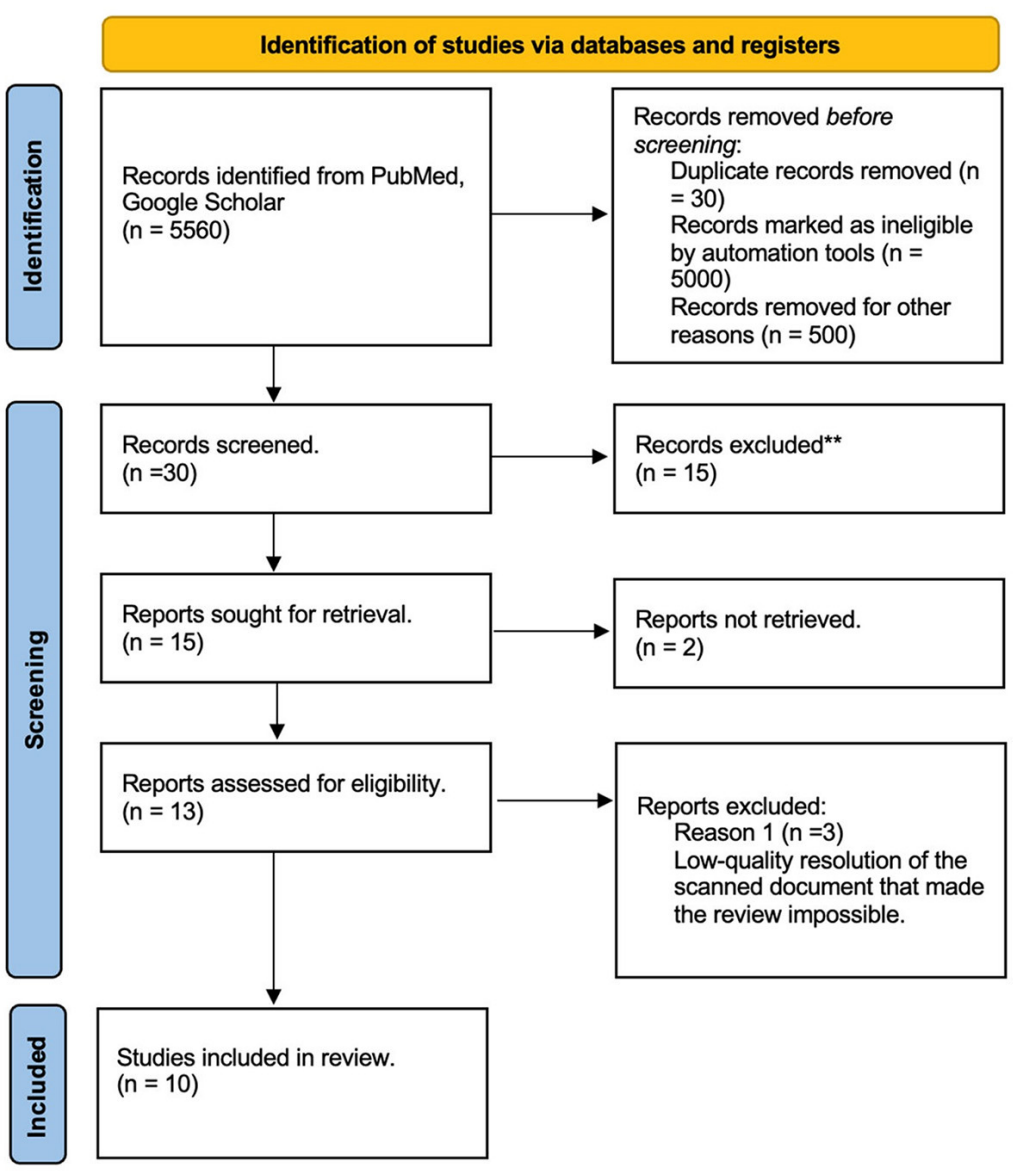
Literature review PRISMA diagram. ^**^ The articles were excluded due to incomplete data, inconclusive findings, language, or full-text not available.

A literature review was performed through two databases, PubMed and Google Scholar. Inclusion criteria were as follows: articles written in English, Spanish, French, or German, including literature until July 2020. Due to the limited availability of Medical Subject Headings (MeSH) terms, the search included the words “Encephalic staining,” “Mulligan protocol,” “Macroscopic brain staining,” “Roberts staining,” “Prussian Blue staining,” and “Alston Staining.” Findable protocols were compared. Used MeSH terms included the following: (“Encephalic” [All Fields] AND (“coloring agents” [Pharmacological Action] OR “coloring agents” [MeSH Terms] OR (“coloring” [All Fields] AND “agents” [All Fields]) OR “coloring agents” [All Fields] OR “stains” [All Fields] OR “stained” [All Fields] OR “staining and labeling” [MeSH Terms] OR (“staining” [All Fields] AND “labeling” [All Fields]) OR “staining and labeling” [All Fields] OR “stain” [All Fields] OR “staining” [All Fields] OR “stainings” [All Fields] OR “staining s” [All Fields] OR “stainning” [All Fields])) OR (“mulligan” [All Fields] OR “mulligan s” [All Fields]) OR “Alston” [All Fields] OR (“brain” [MeSH Terms] OR “brain” [All Fields] OR “brains” [All Fields] OR “brain s” [All Fields]). The analysis followed the Preferred Reporting Items for Systematic Reviews and Meta-Analysis-Scoping Review (PRISMA-ScR) (Figure 1).

### 2.2. Brain specimen preparation

Previously preserved brain specimens in an aqueous 4% of v/v formaldehyde solution were used. These specimens correspond to cadaveric donations acquired for education and research under the institutional research board (IRB) endorsement No. 560-16. Subsequently, samples were cut in 5- and 10-mm width sections, including axial, sagittal, and coronal views. All four staining techniques were carried out by immersion, avoiding touching the structures as much as possible when submerging or moving them unless otherwise indicated. Avoiding this is recommended in all findable protocols. The containers used to immerse the specimens were made of polymer with dimensions of 14.8″ × 10.8″ × 4″, providing a total volume of 8 L. However, it is essential to highlight that the size of the containers will depend on the dimensions of the specimen. The tighter the fit in the container, the smaller the amount of reagent required per total volume. The concentrations at which the different solutions should be prepared are mentioned in the following sections. Inert plastic materials are recommended for the laboratory due to their limited reaction with the reagents.

The reagents used were obtained from local chemical stores following the PubChem (https://pubchem.ncbi.nlm.nih.gov/) and the Computer-Aided Management of Emergency Operations (CAMEO) (https://cameochemicals.noaa.gov) recommendations associated with each reactant.

### 2.3. Staining methods

**Figure 2:**
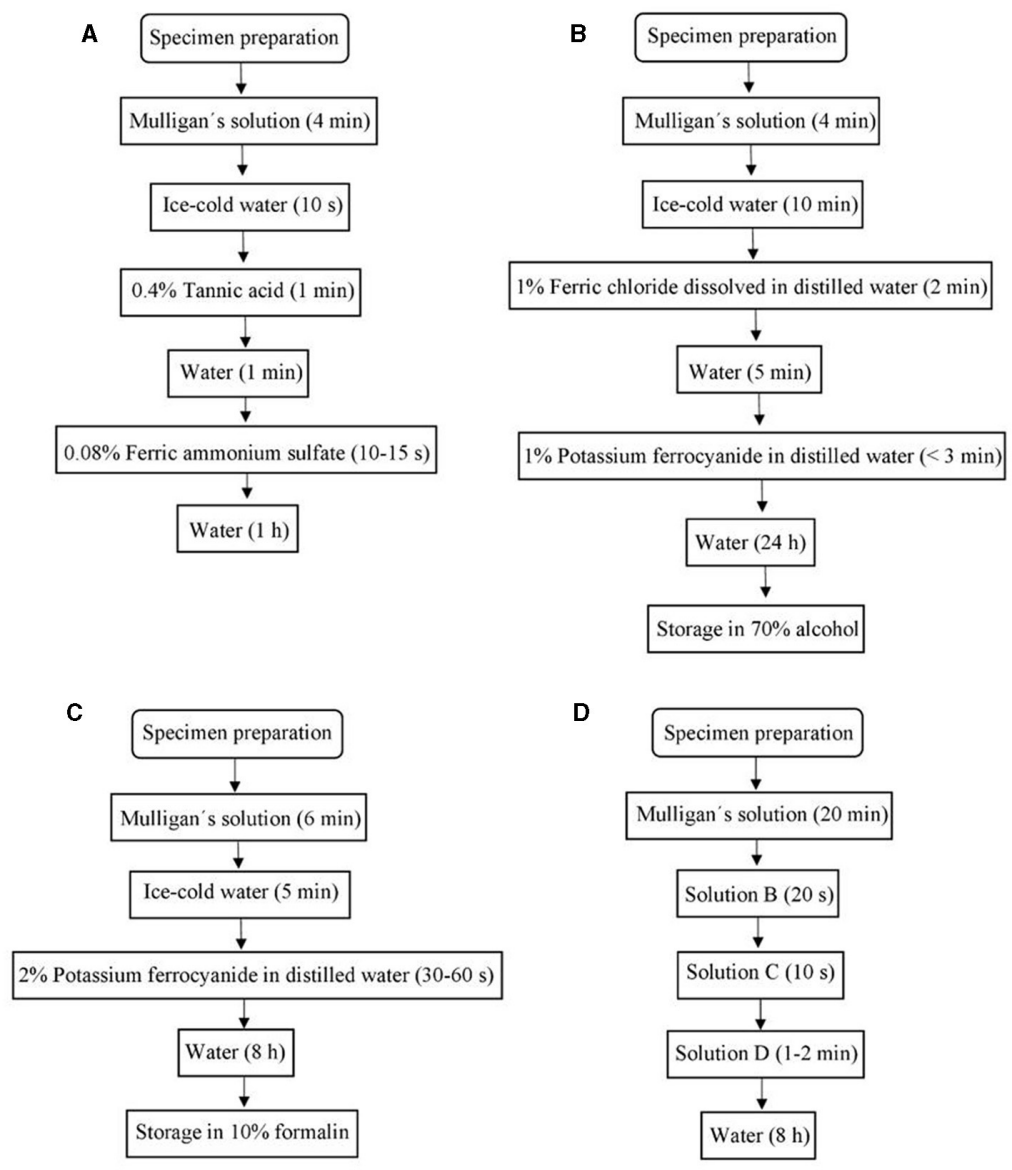
Chart flows of **(A)** Mulligan's stain protocol, **(B)** Prussian Blue stain protocol, **(C)** Roberts's stain protocol, **(D)** Alston's stain protocol.

Sections were divided into four groups depending on the staining protocol to follow: Mulligan (1931), Prussian Blue (1935), Roberts and Hanaway (1969), and Alston (1981); for each one, different conditions, reagent concentration, and impregnation times were evaluated (see Table 1). A summary is shown in Figure 2.

**Table 1:** Gray matter stain, fixation, storage, preparation time, and reagents of different macroscopic brain staining methods used in this manuscript.

**References**	**Staining name**	**Gray matter stain**	**Fixation**	**Storage**	**Preparation time**	**Reagents**	**Original protocol**	**Manuscript protocol**	**Manuscript protocol Result**
**Staining techniques used in this manuscript**									
Alston (1981)	*Alston*	Brick red color	10% formalin v/v	2% aqueous formalin	27.5 min	**Solution A**	1. Solution of: phenol 80% 750 ml, copper sulfate 75 g, hydrochloric acid 15 ml, tap water 15 L (20 min)	1. Specimen preparation	See Figure 3B
						Phenol 80% 750 mL			
						Copper sulfate 75 g			
						Hydrochloric acid 15 mL	2. Solution of: xylene, G.P.R., grade 15 L., polyclens plus 150 ml (20 s)	2. **Solution A:** Mulligan's solution (20 min)	
						Tap water 15 L			
						**Solution B**			
						Xylene, G.P.R, A grade. 15 L	3. Solution of: sodium hydroxide granules 300 g, tap water 15 L (10 s)	3. **Solution B** (20 s)	
						Polyclens plus 150 mL			
						**Solution C**		4. **Solution C** (10 s)	
						Sodium hydroxide granules 300 g	4. Solution of: potassium ferrocyanide 300 g, Water 15 L (1–2 min)		
						tap water 15 L		5. **Solution D** (1–2 min)	
						**Solution D**			
						Potassium ferrocyanide 300 g	5. Water (5 min)	6. Water (8 h)	
						15 L water			
Mulligan (1931) and Gregg (1975)	*Mulligan*	Intense black	10% formalin	70% alcohol	33 h and 18 min	10% Formalin	1. Distilled water (1 h)	1. Specimen preparation	See Figure 3A
							2. Mulligan's solution (4 min)	2. Mulligan's solution (4 min)	
						Tannic acid	3. Ice-cold water (10 s)	3. Ice-cold water (10 s)	
							4.0.4% tannic acid (1 min)	4.0.4% tannic acid (1 min)	
						Iron alum	5. Water (1 min)	5. Water (1 min)	
							6.0.8% ferric ammonium sulfate (10–15 s)	6.0.8% ferric ammonium sulfate (10–15 s)	
						Alcohol	7. Water (1 h)	7. Water (1 h)	
Lemasurier (1935)	*Blue Prussian*	Brillant blue	10% formaldehyde U. S. P.	70% alcohol	24 h and 13 min	2.5 Liters of 4% phenol crystals, 0.5% copper sulfate crystals, 0.125% concentrated hydrochloric acid.	1. Fix in 1:10 formali	1. Specimen preparatio	See Figure 3D
							2. Cut sectiones with a Knife and lubricate with glycerin		
							3. Water (12-24 h)	2. Mulligan's solution (4 min)	
							4. Distilled water/three water changes (1 h)		
							5. Mulligan's solution (2 min)	3. Ice-cold water (10 min)	
						1% Ferric chloride.	6. Ice-cold water (1 min)		
							7.1% ferric chloride in distilled water (2 min)	4.1% ferric chloride in distilled water (2 min)	
							8. Water (5 min)	5. Water (5 min)	
						1% potassium ferrocyanide	9.1% potassium ferrocyanide in distilled water (< 3 min)	6.1% potassium ferrocyanide in distilled water (< 3 min)	
							10. Water (24 h)	7. Water (24 h)	
							11. Preserve in 70% alcohol.	8. Preserve in 70% alcohol.	
Blair et al. (1932)	*Robert*	Light orange	10% formalin	10% formalin	24 h and 20 m	2% Potassium ferrocyanide	1. Perfuse the internal carotid and vertebral arteries with 50 mL of 40% formalin.	1. Specimen preparation	See Figure 3C
							2. Submerge the brain in 10% formalin suspended by the basilar artery (2–4 weeks)		
							3. Cut brains into 4 mm thick sections with a rotary blade. Stack with filter paper between each pair of sections.	2. Mulligan's solution (6 min)	
							4. Water (12 h)		
						Mulligan's phenol solution	5. Mulligan's solution (6 min)	3. Ice-cold water (5 min)	
							6. Ice-cold water (5 min)		
							7.2% potassium ferrocyanide (30–60 s)	4.2% potassium ferrocyanide (30–60 s)	
							8. Water (5 min)	5. Water (8 h)	
							9. Preservation in 10% formalin	6. Storage in 10% formalin	

#### 2.3.1. Mulligan stain

##### 2.3.1.1. Stock Mulligan's solution

Mulligan's solution is a mixture of phenol, iron sulfate, and hydrochloric acid. However, classic Stock Mulligan's solution is always described as using 40 g of 80% of phenol, 5 g of cupric acid, and 1.25 mL of 0.1 M HCl diluted in 1 L of distilled water. To carry out all four staining techniques presented in this article, 10 times the normal quantities (400 g of 80% of phenol, 50 g of cupric acid, 12.5 mL of 0.1 M HCl, and 10 L of distilled water) were used. The solution must be changed if it turns yellow to pale brown (Wu and Kiernan, 2001).

##### 2.3.1.2. Mulligan stain protocol

The brain sections are first immersed in distilled water for 1 h. Second, the specimens are moved to a deep recipient with the Stock Mulligan solution for 4 min at a temperature of 60 to 65°C. Then, the sections are transferred to ice-cold water for 10 s, moved to a deep recipient with a 0.4% of v/v tannic acid solution at room temperature for 1 min, and washed in tap water, and finally, the samples are introduced in 0.08% of v/v ferric ammonium sulfate until the gray substance turns grayish, which takes 10–15 s. The process is stopped with an icy water wash.

#### 2.3.2. Prussian Blue stain protocol

The sections are fixed in a diluted formalin (10% v/v) solution and followed by a continuous flow (wash) in tap water for 12–24 h, continued by distilled water for 1 h. Then, the sections are immersed in a deep recipient with the Stock Mulligan solution at 60–65°C for 4 min. The process is stopped by a quick (no more than 1 min) immersion in ice-cold water.

**Figure 3:**
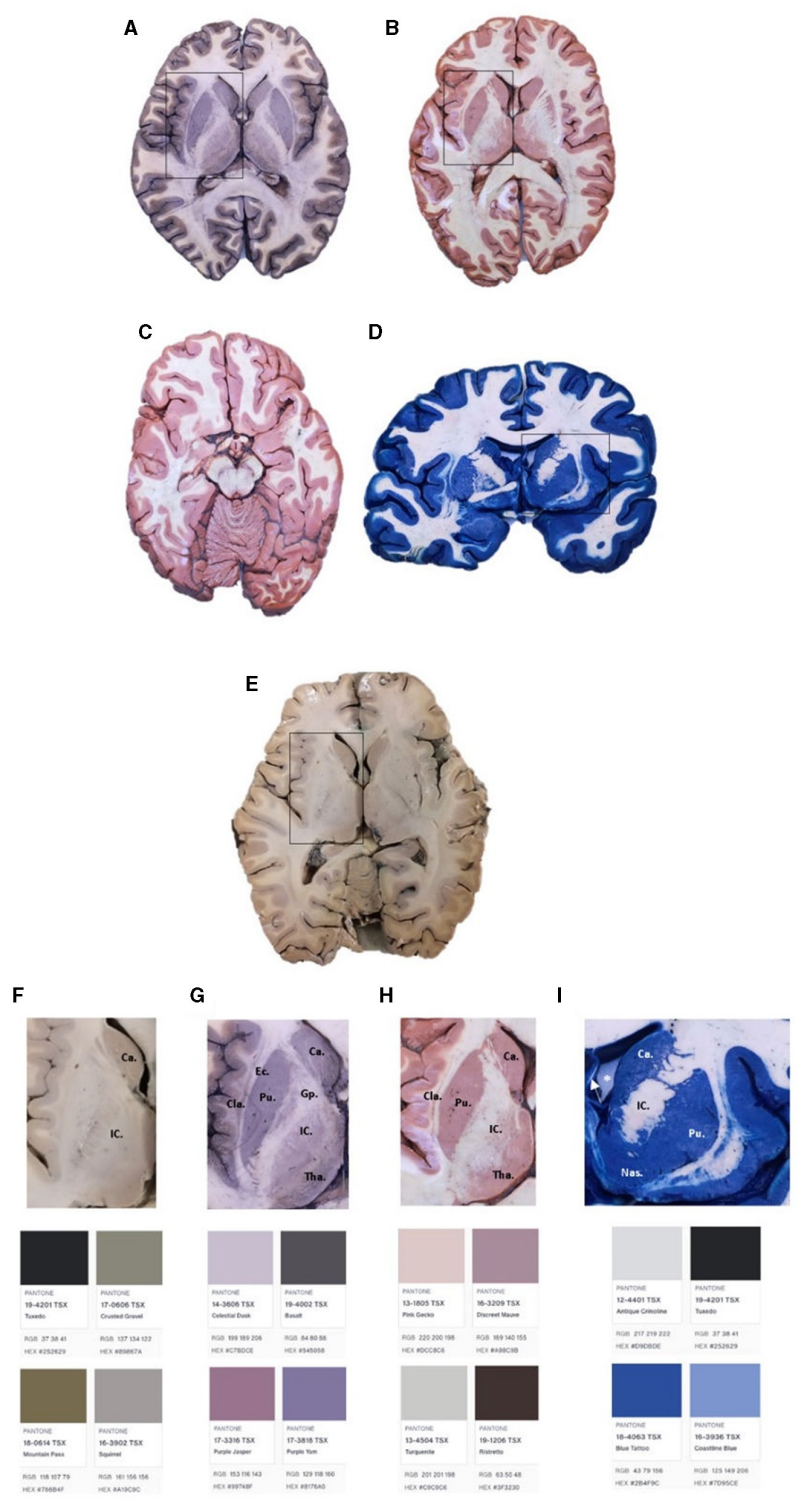
Brain slides with Mulligan's **(A, G)**, Alston's **(B, H)**, Roberts's **(C)**, and Prussian Blue **(D, I)** staining. Brain slide without staining **(E, F)**. Ca, caudate nucleus, Gp, globus pallidum, Tha, thalamus, Pu, putamen, EC, external capsule, IC, internal capsule, Cla, claustrum, Nas, nucleus accumbens septi. *Left lateral ventricle. The arrow represents the septum pellucidum.

The following step (immersion) contains 1% of ferric chloride dissolved in distilled water at room temperature. The brain sections should be kept there for 2 min. Then, we repeat the ice-cold water immersion for 5 min and then dip them in 1% of potassium ferrocyanide solution mixed in distilled water for ~3 min or until a good contrast is obtained. The reaction is stopped using tap water for 24 h as a final step. The final result will show a bright blue-gray matter (see Figure 3D).

#### 2.3.3. Roberts's stain protocol

The suggested section thickness is between 3 and 4 mm. The specimens are immersed in 10% of formalin. A recommendation is to suspend the specimen by the basilar artery for at least 2–4 weeks while still submerged in the formalin. The first step is to wash the specimen in tap water. Then, the sections are immersed in Stock Mulligan solution for 6 min at 60–65°C. The brain slide is then immersed in a cold tap water solution for at least 5 min before being treated with 2% potassium ferrocyanide for 1 min. Sections were washed with tap water and preserved in 10% formalin.

#### 2.3.4. Alston's stain protocol

First, the specimens were immersed in solution A for 10 min, then in solution B for 20 s, solution C for 10 s, then solution D for 1–2 min, or until the desired color was obtained. Finally, the reaction is stopped by a tap water immersion for at least 8 h. The final result is a reddish color in the gray matter (see Figure 3B).

##### 2.3.4.1. Solutions

Quantities for each of Alston's staining solutions are presented below.

2.3.4.1. Solution A: stock Mulligan solution.
2.3.4.2. Solution B: this solution has 15 L of xylene, G.P.R, A grade, and 150 mL of Polyclens.
2.3.4.3. Solution C: this solution contains 30 g of sodium hydroxide granules and 15 L of tap water.
2.3.4.4. Solution D: this solution contains 300 g of potassium ferrocyanide and 15 L of tap water.

### 2.4. Preservation and plastination

For long-lasting preservation, a plastination process with S-10 silicone (Biodur^®^) is processed following the protocol of our laboratory.

### 2.5. Likert scale survey

A voluntary survey was conducted among second-year medical students during a neuroanatomy class, examining both stained and non-stained brain sections. Given its anonymized nature, the survey received an IRB waiver. To ensure that the students felt no pressure to participate, they were informed at the survey's outset that their responses would remain completely anonymous and would have no impact on their grades, even if they chose not to answer. The anatomy class coordinator independently verified that no coercion had been exerted on the students. Data collection was carried out by a member of the anatomy research group who was not among the main authors, and the transfer of data to an Excel spreadsheet was performed by another research group member who was not involved in the survey.

The survey consisted of three Likert scale questions and one open question regarding their perspective after using these stained brain sections compared with non-stained sections. Each Likert scale question was ranked from 1 to 4, 1 being —“100% disagree with the affirmation,” 2—“Mostly disagree with the affirmation,” 3—“Mostly agree with the affirmation,” and 4—“100% agree with the affirmation.” The “open answer” question was “What is your personal opinion about stained sections and their impact on your learning experience?” Forty-three participants voluntarily answered the survey.

## 3. Results

### 3.1. Historical review

In 1931, Mulligan, a surgeon with expertise in chemistry, made significant advancements in macroscopic staining techniques for gray matter (Mulligan, 1931). Using substances that dissolved the lipids of neurons' myelin sheath, Mulligan successfully delineated the gray matter, which was then stained black using tannic acid and ferric alum (Mulligan, 1931). In 1935, Lemasurier combined Sincke's Prussian Blue stain from 1926 with the Mulligan solution, finding that tannic acid had a similar effect as ferric chloride in enhancing brightness with Prussian Blue (Lemasurier, 1935). Plastification was introduced by Kampmeier, Haviland, and Hospodar around 1949 to preserve the sections (Kampmeier and Haviland, 1948; Kampmeier and Hospodar, 1951). In 1959, Hewitt developed a Sudan staining technique that provided better differentiation between gray matter (GM) and white matter (WM) than Mulligan's stain, with GM appearing as bright red and WM as pale pink (Hewitt, 1959).

In 1969, Roberts and Hanaway from the University of Virginia Department of Anatomy published a staining method using copper sulfate and potassium ferrocyanide, resulting in a reddish color (Barnard et al., 1949; Roberts and Hanaway, 2009). Alston improved the protocol in 1981, achieving a better distinction between GM and WM (Alston, 1981). In 1971, Augulis developed a vascular perfusion staining method for observing brain and spinal cord structures in animals, allowing simultaneous staining and fixation in the same solution, thus facilitating efficient evaluation of these structures (Augulis and Sigg, 1971).

#### 3.1.1. Twenty first century: the present and future utilities

In 2008, Loftspring et al. proposed a copper (II) technique using 1% of phthalocyanine-tetra sulfonic acid tetrasodium salt in water for 2 h, followed by acetic acid treatment, without prior treatment with Mulligan's solution, for research and teaching purposes, yielding better results than the previous Mulligan solution treatment (Loftspring et al., 2008). Finally, Cruz et al. (2020) aimed to establish a new protocol for the Prussian Blue stain, which is essential for teaching in laboratories and medical schools. Their protocol was similar to the one described in this article. However, they stored the samples in 4% of formaldehyde with 0.5 cm^3^ of hydrochloric acid per 1 L of formalin, and the immersion time in potassium ferrocyanide was reduced to only 10–15 s (Cruz et al., 2020).

### 3.2. Staining's results

Table 1 summarizes the classical techniques found in our review, including data related to the origin of the method, expected gray matter color, preparation time, reagents, fixatives, and the conservation method, as well as the original protocol of the technique and the protocol that we carried out in this article together with the result that we obtained. Table 2 summarizes the rest of the methods found in the literature review, but that were not performed for this article. The most used fixation method corresponded to 4% of formalin. Throughout history, 70% alcohol has been the most widely used conservation method. Most staining protocols take up to 2 days to perform.

**Table 2:** Gray matter stain, fixation, storage, preparation time, and reagents of other macroscopic brain staining methods.

**References**	**Gray matter stain**	**Fixation**	**Storage**	**Preparation time**	**Reagents**
**Other staining techniques**					
Braak (1978)	Brilliant Blue	4% formalin	Formalin-hardened gelatin	22–34 h	4% formalin
					Performic acid: 100 cc H2O2 (30%) to 900 cc formic acid (98–100%)
					0.1 g Astra blue in 1,000 cc distilled water and add 1 cc HCl (37%)
Brown (1939)	Chocolate-brown	10% formalin v/v	10% formalin	20.4–32.4 h	10% formalin
					Carbolic acid
					1% lead nitrate
					1% silver nitrate
					5–10% ammonium sulfide
					1% solution of crystal violet
Blair et al. (1932)	Dark gray	10% formaldehyde U. S. P.	70% alcohol	24 h and 21 min	5% sodium sulfide
					Cobalt nitrate
					70% alcohol
					Formaldehyde
Blair et al. (1932)	Yellow	10% formaldehyde U. S. P.	70% alcohol	24 h and 20 min	1% lead nitrate
					5% potassium iodide
					70% alcohol
					Formaldehyde
Blair et al. (1932)	Dark purple	10% formaldehyde U. S. P.	70% alcohol	48 h and 3 min	Soluble starch solution.
					Solution of iodine in potassium iodide.
Wu and Kiernan (2001)	Light blue	4% neutral buffered formaldehyde	0.5% acetic acid	36 min	Copper (II) phthalocyanine tetra sulfonic acid (CPTS) solution
					Tetrasodium salt
					Glacial acetic acid 5 ml
					Mulligan's solution

Figure 3 shows the brain sections after the four staining techniques were performed. Sections A, B, D, and E were approached at the level of the basal ganglia. Figure 3A shows Mulligan's staining results in an axial section at the level of the basal ganglia. Figure 3B exhibits Alston's staining in an axial slice at the same level. Figure 3C presents Roberts's staining in an axial brain section at the midbrain level. Figure 3D shows Prussian Blue staining in a coronal brain slice at the third ventricle level. Finally, Figure 3E represents an axial section without staining at the basal ganglia level. Figures 3F–I shows a close caption to basal ganglia and colorimetric analysis (generated by Pantone Connect: connect.pantone.com).

All protocols showed excellent deep brain structure differentiation. Mulligan protocol (Figure 3A) showed a darker contrast than the non-stained section (Figure 3E). The basal ganglia complex is easily seen, and the differentiation between the external capsule and the claustrum shows a considerable contrast enhancement. Compared with Alston's and Roberts's staining (Figures 3B, C), which appear more in the order of pink and red, as classically described (Figure 3H), the classic Mulligan staining presents with less contrast, more in the gray/purple gradient (Figure 3G). Finally, a comparison between Prussian Blue (Figure 3D) shows a more significant difference than the rest of the stainings, appearing in the blue range (Figure 3I) as expected.

### 3.3. Student survey results

Students' responses corresponded to 64% of the neuroanatomy class (43/67). The survey was divided into three *Likert scale questions ranked from 1 to 4, 1 being —“Disagree 100% with the affirmation,” 2—“Mostly disagree with the affirmation,” 3—“Mostly agree with the affirmation,” and 4—“Agree 100% with the affirmation.” The “open answer” question was, “What is your opinion about stained sections and their impact on your learning experience?”*

**Figure 4:**
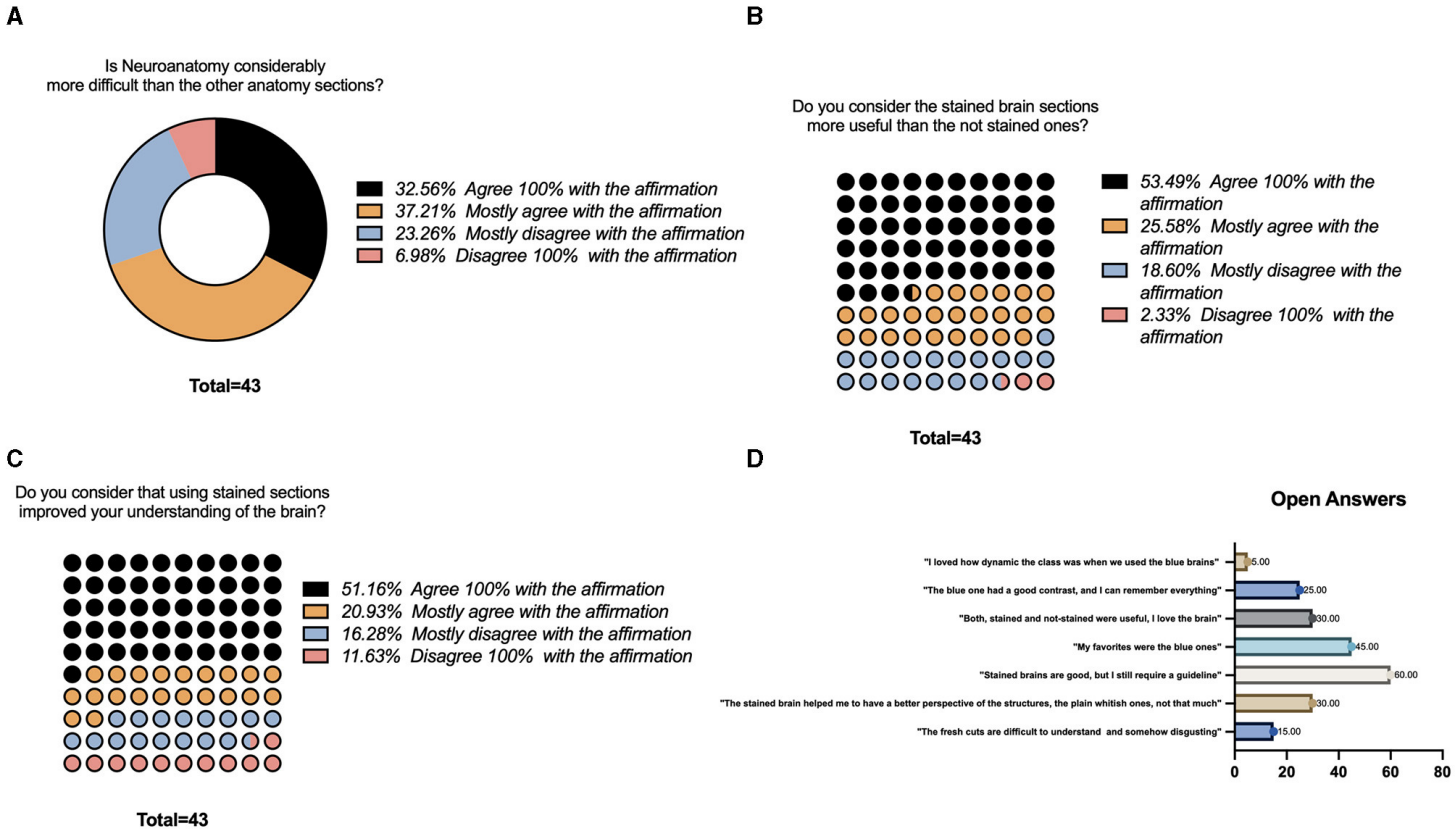
Survey results. **(A)** Likert-scale question regarding neuroanatomy complexity compared to other sections of the class. **(B)** Likert-scale question regarding usefulness of the stained sections while learning neuroanatomy. **(C)** Likert-scale question regarding improvement while learning neuroanatomy using the stained sections. **(D)** Open questions regarding the use of the stained sections.

Forty-three participants voluntarily answered the survey. For the first question regarding their perception of the neuroanatomy section compared with other anatomy sections, 32.56% agreed 100% that the class was more difficult, followed by 37.21% who partially agreed. Only 6.98% completely disagreed with the affirmation (Figure 4A). For the second question regarding their perspective on how useful the stained brain sections were compared with the non-stained ones, 53.49% believed that they were more helpful, followed by 25.58% who partially believed that they have better applications, and 2.33% did not think they were more valuable (Figure 4B). Finally, for the third question whether their experience learning neuroanatomy improved with stained sections, 51.16% agreed 100% with this affirmation, and 11.63% did not believe that using stained sections helped their understanding of the brain (Figure 4C).

Open answers regarding their perspective were summarized by frequency in Figure 4D. Some of the common opinions included the following: “*The fresh cuts are difficult to understand and somehow disgusting” (15 %), “The stained brain helped me to have a better perspective of the structures, the plain whitish ones, not that much” (30%), “Stained brains are good, but I still require a guideline,” (60%), “My favorites were the blue ones (45%)” “Both, stained and no-stained were useful” (30%), “The blue one has a good contrast and helped me to identify all the structures, I remember everything” (25%), “I loved how dynamic the class is when we use the sections, particularly the blue ones (5%).”*

## 4. Discussion

### 4.1. Historical review

As a first approach in neuroanatomy, structure identification can be challenging without any enhancing staining, particularly during dissection. Macroscopic stains are essential in studying deep brain cerebellar nuclei, basal ganglia, and tracts (Baeres and Møller, 2001; Punnarat and Ornsiri, 2014). Over the years, different macroscopic brain tissue staining techniques have been described (see Tables 1, 2). These techniques coincide with intending to increase the contrast between WM and GM. These improvements did facilitate the identification of nuclei, along with the delimitation of the cerebral cortex and cerebellar fibers of WM (Hewitt, 1959; Braak, 1978). However, these techniques have been disused nowadays due to advances in microscopic staining, molecular biology, and advanced neuroimaging techniques (Jeans and Esiri, 2008). Nevertheless, macroscopic stains for brain tissue are still valid as a tool for neuroanatomical education, given the practicality of their use and the need to facilitate the distinction of neuroanatomical structures for the student (Loftspring et al., 2008). In 1933, Green explained how specimens prepared with macroscopic stains had great usefulness in teaching; these stains could last for years without significant changes, facilitating the study of species over time (Green, 1933).

In the 16th century, Andreas Vesalius (1514–1564), the father of modern anatomy, was the first to describe white matter and gray matter as brain components (Schmahmann and Pandya, 2007; Boullerne, 2016). In 1543, Vesalius published his famous illustrated treatise on anatomy, De humani corporis fabrica libri septem (on the structure of the human body). Vesalius distinguishes the yellowish and soft substance from the hard white substance for the first time in the brain (Schmahmann and Pandya, 2007).

In 1833, Ehrenberg described the neuron as a functional unit of the nervous system (NS), and 5 years later, Johannes Purkinje performed a broad cytoarchitectural characterization of different brain and spine regions (Swanson, 2007). However, it was not until 1854 that the first brain tissue stain by Joseph von Gerlach was incidentally developed using a carmine extract on cerebellum sections (Wickens, 2014). Carl Weigert, one of the most influential researchers in the macroscopic brain stain development history, developed the myelin sheath stain between 1882 and 1891 (Sammet, 2008). This staining method sought to overcome the limitations of the predominant carmine stain of the time, which was developed by Joseph Gerlach (Sammet, 2008). In 1882, and during an epidemic of smallpox in Breslau in 1870–1871, the Weigert stain served for infectious analysis and later, with some modifications, for detailed cortical analysis (Sammet, 2008). Between 1906 and 1909, Christfried Jakob focused on understanding the frontal lobe, from childhood to old age, its philology, and its pathology (Théodoridou and Triarhou, 2012). In some of his sketches, it is possible to observe the differences he makes between the cortex and the cerebral parenchyma, showing the necessity that existed in the difference between these two crucial brain areas (Théodoridou and Triarhou, 2012).

In the second decade of the 20th century, Landau made the first report in Switzerland on a chemical method for macroscopically differentiating white matter (WM) from gray matter (GM) in brain sections using Prussian Blue as a dye (Lemasurier, 1935). Sinke later replicated this method in 1926 (Lemasurier, 1935). The protocols of Landau and Sinke consisted of immersing sections fixed in formol in a ferric chloride solution, washing the sections, and then immersing them in ferrocyanide of potassium. Hence, their results were similar: GM turned dark blue, and the WM became lighter blue (Lemasurier, 1935). The contrast between the two components increased slightly. However, staining faded over time. Mainland in 1928 modified the technique and increased the staining duration (Mulligan, 1931).

### 4.2. Staining protocol modifications

The four macroscopic staining protocols described in the Materials and methods section showed promising deep brain structure contrast and differentiation results. These macroscopic staining methods were chosen given their remarkable ease of preparation, their vast and distributed use in the study of neurosciences, and the observed difference between WM and GM reported in the literature. Small changes were made to the original protocols (see Table 1). However, there were no changes in the type and quantity of reagents.

In our experience, preservation with ethanol is feasible; however, when not managed properly, the fibers are prone to extreme desiccation, changing the stain adherence. Paraformaldehyde in different concentrations (4–10%) did not modify the result; however, in our regular practice, we do not use either paraformaldehyde or ethanol as the primary source of fixation in brains. We developed a solution that was discussed in the article by Rueda-Esteban et al. (2017). In this study, specimens preserved in a 4% formaldehyde solution, a formaldehyde-free solution (FFS), or initially fixed with a 10% formaldehyde solution and later embalmed with FFS were used, obtaining similar results in staining with the different formaldehyde solutions.

Regarding the thickness, the results are similar if a window between 0.5 and 1 cm is maintained. Thinner slices will result in over-saturation of the color. In addition, a more considerable difference between GM and WM can be seen in the stained sections (Figures 3A–D) compared with the difference seen in Figure 3E. Structures such as the globus pallidus, the putamen, the internal capsule, the external capsule, and the thalamus can also be seen much better in Figures 3A, B compared with Figure 3E. This demonstrates the value of macroscopic brain stains in identifying specific brain structures, some of which are very useful in teaching and researching neuroscience.

Macroscopic stains have been well used for many years as a practical and effective tool for teaching neuroanatomy (Lemasurier, 1935). Staining techniques are essential for the student's accurate learning and the development of mental tools that will allow him/her to better understand neurological pathologies and interpret diagnostic images (Ccorahua Rios et al., 2020). The specimens dyed with Prussian Blue in the article have similar results. However, there is a marked difference in the color obtained for Roberts's staining. In comparison to our pale red, the result is a golden staining of the gray substance. However, there is still a big difference between WM and GM in both cases. Better results in differentiating WM and GM can foster a better understanding of the complex anatomical relationships of the human brain in students, contributing to the development of spatial intelligence that will allow them to position themselves better and more quickly in diagnostic images used daily in medical practice, seeking better and more accurate diagnoses.

Comparing the results of Loftspring et al. (2008) with those obtained in this study, there is a noticeable difference between gray and white matter; however, these differences are enhanced when we use the blue Prussian Blue protocol. It is necessary to clarify that a change was made to the original Prussian Blue stain in our protocol. In the original protocol, the pre-treatment with Mulligan's solution was 2 min (Lemasurier, 1935; Braak, 1978), but we did it for 4 min, obtaining a darker blue color and increasing the differentiation between gray and white matter. In another article, the results obtained by Cruz et al. (2020) show a more precise tone in the Prussian Blue stain compared to ours. This can be explained by the subtle differences between the protocol they carried out and the one followed for this study. Although the differences between the tones obtained and those reported in other articles are small, they should be evaluated when using macroscopic stains as a teaching method in neuroanatomy.

### 4.3. Plastination as a long-lasting alternative for color preservation

Combined with plastination techniques, macroscopic staining techniques offer an alternative for conserving specimens, considering the high educational demand and the poor supply of brains for study (Baeres and Møller, 2001). Applying hand plastination techniques to different macroscopic staining methods is especially useful given the low equipment cost and ease of plastination (von Hagens et al., 1987).

Despite the staining protocol, the color does not fade after 5 years of use. This tendency is maintained regardless of the preservation method of choice (formaldehyde 4–10%, ethanol 70%, vacuum-sealed plastic bags, or silicon/resin plastination). Nevertheless, as educational specimens, continuous usage may generate damage to the specimens. Therefore, from our experience, plastinated models exhibit the best shelf-life. The S10 plastination method was the most helpful combination with macroscopic staining methods in a study, suggesting that, of the methods used, the Alston staining method was the one that gave the best results (Suriyaprapadilok and Withyachumnarnkul, 1997). Combining the staining methods presented in this article with plastination protocols would be very useful for teaching and research in neurosciences.

An article published by Punnarat and Ornsiri compared Mulligan's, Alston's, and Prussian Blue's staining of dog brain slices before plastination (Punnarat and Ornsiri, 2014). Comparing the results obtained in their article and the results obtained in this article, we can observe a much more marked difference between the WM and GM in our sections. As the staining protocol used in both articles was practically the same, one can think that, given the nature of the dogs' brains, the results may differ depending on whether a human brain is used. This highlights the importance of an excellent staining technique and a suitable conservation method to maintain human specimens.

Functionally, anatomical knowledge of the structures found in the midbrain is essential for fields such as neurosurgery (Alho et al., 2011). dos Santos et al. (2019) published an article describing the anatomical relationships with the internal globus pallidus and correlated it with previous anatomical studies. One method used to correctly place the electrodes in surgery was the stereotactic atlases, which were based on anatomical studies with postmortem macrocephalic stains to be created. This same idea can be applied to cases of deep brain stimulation in patients with Parkinson's disease as the correct placement of the electrodes, with prior knowledge of the anatomical distribution of the subthalamic nuclei, is essential for good outcomes (Alho et al., 2011).

## 5. Limitations

First, the most significant study limitation was the antiquity of the documents, which is also related to the difficulty encountered when establishing the search terms since many of the articles were outside conventional databases. Second, students' responses corresponded to 64% of the neuroanatomy class (43/67); this sample size is a limitation as it may not be representative, compromising the validity of the obtained results. Finally, physical variables such as pressure, humidity, and temperature at which the stains presented in the article could influence the results obtained and their differences from the effects of other articles.

## 6. Conclusion

Despite the increasing use of microscopic stains for brain study, macroscopic brain stains continue to be a valuable studying, learning, and researching tool in neuroanatomy. Further research on the stained specimens' utility as learning tools is needed. These techniques allow the creation of several brain-slide databases that can allow a three-dimensional comprehension of the encephalic structure. Furthermore, understanding the basic chemistry principles related to the techniques opens new doors for innovative designs, such as in electronic microscopy.

## Author contributions

GV-G proposed the main idea for the protocol, supported the literature review and initial writing, and contributed to the article's design and writing. LF proposed the main idea for the protocol, supported the literature review, initial writing, staining process, and survey, and contributed to the article's design and writing. AR proposed the main idea for the protocol, supported the literature review, performed the staining, and contributed to the writing of the article. PQ-P wrote the main idea for the protocol, supported the literature review, and performed the staining. RR-E supervised the staining and quality control, contributed as a secondary reviewer in the literature search, and corrected the final version of the manuscript. All authors contributed to the article and approved the submitted version.
